# P01 Adult-onset chronic recurrent multifocal osteomyelitis – case series of four adult patients in Liverpool University Teaching Hospitals Foundation Trust

**DOI:** 10.1093/rap/rkae117.032

**Published:** 2024-11-05

**Authors:** Mark Tern, Kashif Malik, Jennifer Christie

**Affiliations:** Liverpool University Hospital, Liverpool, United Kingdom; Liverpool University Hospital, Liverpool, United Kingdom; Liverpool University Hospital, Liverpool, United Kingdom

## Abstract

**Introduction:**

Chronic recurrent multifocal osteomyelitis (CRMO) is a rare inflammatory bone disorder. Classically known as a childhood disorder and diagnosis of exclusion, it characteristically presents with relapsing sterile bone inflammation affecting the metaphysis of lung tubular bones, shoulder girdle, clavicle, vertebral column or bony pelvis. Links with autoimmune conditions have been suggested.

Evidence of adult-onset cases (age >18 at diagnosis) remains sparse and sporadic, with mostly individual case reports on searches. We present a case series of four patients with adult-onset CRMO in Liverpool, with varying responses to bisphosphonate treatment and no clinical features of SAPHO.

**Case description:**

We list four cases of adult patients with CRMO.

Case 1: 53-year-old female presented in 2023 with a 10 year history of on-off chest pain isolated to her sternum, and back pain since 2021. MRI confirmation of bone marrow oedema. Bone biopsy normal. Treated with zoledronate. MRI following treatment suggested some improvement but patient remained in pain.

Case 2: 24-year-old female presented in 2019 to services with a 10 year history of worsening back pain. Medical background of asthma, type 1 diabetes mellitus, primary biliary cirrhosis and psoriasis. MRI suggestive of persistent pre-sacral soft tissue and sacral oedema since 2015. ANA, Anti-CCP, ENA and dsDNA were negative. Bone biopsy done was positive for inflammation. Pain improved with pamidronate.

Case 3: 31-year-old male presented in 2022 with 14 years of relapsing pains and swelling in left clavicle and improvement with NSAIDs. Initially presented via orthopaedic services who arranged an MRI scan that demonstrated oedema of his left clavicle. Received zoledronate infusion with improvement in pain.

Case 4: 45-year old-female presented in 2024 with eight year history of chronic right hip and right sacroiliac joint pain. Persistent lower back pain, central right sided with radiation down right knee to her knee. Examination showed tenderness over her lumbar spine and sacroiliac joints. PET-CT done showed avid uptake in L3/4, with subtle lucencies in left frontal bone, right femur and right sacrum. Treated as CRMO with pamidronate with a positive response.

All patients received bisphosphonate therapy and varied response to treatment was seen. Two of the four patients had an excellent response with complete resolution of symptoms. One patient has had initial positive response to treatment with resolution of pain but is still planned for more bisphosphonate cycles. One patient had no response to treatment.

**Discussion:**

Current literature searches describe CRMO mostly in the paediatric populace, with peak incidence aged 10. Most cases of CRMO are diagnoses of exclusion, hence leading to diagnostic delays as differentials such as malignancy, infection, and histiocytosis are first excluded. The Bristol review study suggested a mean time of 15 months to diagnosis in the paediatric populace.

We highlight here our findings that CRMO can also present within the adult populace. Typically in adults when similar symptoms arise, SAPHO syndrome (synovitis, acne, pustulosis, hyperostosis and osteitis) is considered as a differential. Our patients, whilst having features on imaging suggestive of bone inflammation, did not have any features of SAPHO syndrome. Our findings are corroborated by the Eurofever International Registry study that enrolled 485 patients and showed adult-onset disease in 6.3% of their cohort, accounting for 31 cases.

Our patients were all treated with bisphosphonate therapies. Currently, bisphosphonate therapies are often agreed as a first line treatment approach. Some reports exist of beneficial effects in the use of corticosteroids, DMARDS - particularly sulfasalazine and methotrexate - and biologic treatments (anti-TNF agents), though no common consensus guideline for treatment of CRMO within the UK exist at the moment to our knowledge.

We feel the varied clinical response of our patients highlights a need for more research to be targeted towards this area.

**Key learning points:**

Learning points are as follows:

• Few cases of adult-onset CRMO are reported in the UK. Most are single site case reports or case series of paediatric patients.

• This cases series is the largest adult-onset cohort in Europe from a single hospital site.

• Bisphosphonates are used first line - typically pamidronate.

• Corticosteroids, DMARDs (SLZ/MTX) and biologic treatments (anti-TNF agents) have been reported to be effective in some single case reports.

• Additional research is required for a national consensus guideline for the treatment of CRMO, including additional management options for those patients that fail first line treatment (bisphosphonate).

